# A Novel Vitamin E Adjuvanted Injectable *Bordetella bronchiseptica* Vaccine Is Safe and Efficacious in Dogs

**DOI:** 10.3390/vaccines14040344

**Published:** 2026-04-14

**Authors:** Beth Bruton, Pieter A. W. M. Wouters, Ian Tarpey, Jacqueline Pearce

**Affiliations:** MSD Animal Health, 5831 Boxmeer, The Netherlands

**Keywords:** *Bordetella bronchiseptica*, vaccination, vaccine efficacy, puppies, injectable, fimbriae

## Abstract

**Background/Objectives:** *Bordetella bronchiseptica* is a Gram-negative bacterium that, either acting alone or in concert with other bacterial or viral pathogens, is a major cause of the canine infectious respiratory disease (CIRD) complex in dogs. Most currently available vaccines are given intranasally or orally and, whilst providing satisfactory reduction in disease severity, can be difficult to use especially in aggressive or anxious dogs. Whilst a small number of injectable *B. bronchiseptica* vaccines have been developed, little is known about their characteristics with regard to the age at first vaccination, the onset of immunity, duration of immunity, induction of antibody responses, concurrent use with the core vaccines used in most dogs, efficacy in the face of maternally derived antibodies (MDAs) or existing immunity and safety in pregnant animals. Here we describe the development of a safe and efficacious injectable *B. bronchiseptica* vaccine that utilises a novel process to purify fimbriae. **Methods:** The fimbrial antigen was formulated with a vitamin E-based oil-in-water adjuvant known to be safe in dogs (Nobivac^®^ Respira Bb). To evaluate dose response, thirty-nine naïve 5–6-week-old Beagle puppies were allocated to four groups and vaccinated subcutaneously with Nobivac^®^ Respira Bb at 69 U, 25 U, and 7 U (with a booster at two weeks). All groups were challenged with *B. bronchiseptica* two weeks after the booster. To evaluate the onset of immunity at 5–6 weeks of age, twenty-one naïve Beagle dogs were split into two groups: group 1 received Nobivac Respira Bb (88 U/dose) plus Nobivac DHPPi and Nobivac L4; group 2 received DHPPi and L4 only. Both groups were challenged with *B. bronchiseptica* two weeks after the second vaccination. Safety in pregnancy was evaluated by vaccinating pregnant dams and monitoring whelping outcomes and puppy health. Protection in puppies with maternally derived antibodies (MDAs) was studied in 28 pups (11 MDA-negative and 17 MDA-positive from vaccinated and unvaccinated dams). Pups were vaccinated at 5–6 weeks; one group remained unvaccinated to monitor MDA kinetics. All puppies were challenged with *B. bronchiseptica* at 19 weeks, after MDAs became undetectable. Serology was monitored throughout; daily clinical observations and nasal swabs post-challenge assessed protection and bacterial shedding. **Results:** Nobivac Respira Bb (MSD Animal Health), was safe for use in 5–6-week-old puppies alongside other Nobivac core canine vaccines without vaccine interference. The vaccine has an onset of immunity of two weeks and significantly reduces both the clinical signs of *B. bronchiseptica*-induced disease and bacterial excretion into the environment. Furthermore, the vaccine is equally efficacious in puppies with maternally derived antibodies derived from vaccinated dams and can be used safely in pregnant bitches. **Conclusions:** This vaccine represents a convenient, safe and efficacious alternative to vaccines delivered via the oral or intranasal routes and is a positive addition to the range of vaccines targeted at reducing disease induced by *B. bronchiseptica*.

## 1. Introduction

*Bordetella bronchiseptica* is a Gram-negative bacterium, a primary pathogen capable of causing upper respiratory tract disease in a number of mammals, including pigs, rabbits, cats and dogs. In rare circumstances, humans, especially those who are immunosuppressed, can also become infected and manifest signs of respiratory disease [1]. Canine Infectious Respiratory Disease (CIRD) can either be caused by *B. bronchiseptica* alone or in combination with other bacterial or viral pathogens [2,3]. The severity of the disease can largely be moderated by use of a range of available vaccines, including monovalent *B. bronchiseptica* vaccines or those including canine parainfluenza and canine adenovirus type 2. For the most part these vaccines are either delivered intranasally or orally and in addition to reducing disease severity can lower the amount of *B. bronchiseptica* shed into the environment or to other animals [4,5,6,7,8,9]. Most of these vaccines tend to include a live attenuated strain of *B. bronchiseptica*, which, in the case of intranasally vaccinated animals, can give a rapid onset of immunity within as little as 72 h following a single dose [8]. Intranasal vaccines have also been shown to induce an IgA response in the respiratory tract [9]. Vaccines given orally are also effective but may have a slower onset of immunity compared to intranasal vaccines [8,10]. However, for a proportion of dogs, particularly aggressive or anxious animals, vaccination via these routes is not practical [11]. For this reason, injectable vaccines are often preferred [12], and these are usually given via the subcutaneous route, the most common vaccination route for core canine vaccines.

However, with these particular *Bordetella* vaccines, little is known about their characteristics with regard to the age at first vaccination, the onset of immunity, induction of antibody responses, duration of immunity, use in the face of maternally derived antibodies (MDAs) or existing immunity and safety in pregnant animals. Furthermore, it has been demonstrated that these vaccines are unlikely to induce local IgA responses [9], which may affect their efficacy at the site of infection, particularly in respect of reducing bacterial shedding. For this reason, we aimed to develop a vaccine which could be given conveniently by subcutaneous vaccination alongside routine core vaccines at an early age but that would also be efficacious in the face of MDAs, have a characterised duration of immunity and be safe in pregnant animals. In order to achieve these features, it is important to utilise an adjuvant known to be safe in dogs and to identify an appropriate antigenic component of the vaccine, the interaction between these two components being crucial to the safety and efficacy balance of the vaccine with any significant reactivity being unacceptable to both veterinarian and owner.

There are a range of vaccine adjuvants that can be used in different animal species, including those based on aluminium salts, block polymers, water-in-oil adjuvants, oil-in-water adjuvants, saponins and TLR agonists [13]. These vary in their ability to induce different types of immune response, including both innate and adaptive immunity, onset of protective immunity and the duration of protection. Careful consideration needs to be given regarding the use of these adjuvants to avoid adverse reactions in animals especially when considered in relation to the antigen in the vaccine formulation. In view of that, an oil-in-water-based adjuvant containing a final concentration of 7.5% dl-α-tocopheryl acetate, a form of vitamin E, was developed (Nobivac Respira Bb, MSD Animal Health). The adjuvant is known as Diluvac Forte and is used in various swine vaccines, including Porcilis AR-T (MSD Animal Health), which contains *Bordetella bronchiseptica* [14] and a further derivative of this adjuvant, designated Micro Diluvac Fortasol, which is known to be safe in dogs.

Although the exact mechanism of action of oil-in-water adjuvants is unclear, it is known that they do not work via a depot effect with slow release of antigen as seen in water-in-oil adjuvants. Indeed, oil-in-water-based vaccines do not persist at the site of injection but are thought to induce immunity by attracting cells of the immune system, including neutrophils and monocytes, to the site of injection [15]. α-Tocopherol, known to possess antioxidant activity, is thought to have immunomodulatory properties, and its safety profile is such that it is used in human oil-in-water vaccines such ASO3, part of the pandemic influenza vaccine Pandemrix^®^ [16]. Interestingly the omission of α-tocopherol in AS03 has been shown to negatively modify the profile of the innate immune response and result in a lower antibody response [17]. In addition, α-tocopherol has also been shown to reverse the suppressive effect of T cell activation and thus enhance the immune response to immunisation [18,19].

As previously described, the *B. bronchiseptica* vaccines delivered intranasally or orally are based on live attenuated bacteria. Injection of these live bacteria via parenteral routes can be highly dangerous, requiring treatment with antibiotics [20]; thus, for an injectable vaccine a safe bacterial subunit component is required. However, preparing subunit vaccines against Gram-negative bacteria is challenging as it must take into account the complex structure of the cell wall of these organisms. In particular the challenge is to remove endotoxins such as lipopolysaccharide (LPS) sufficiently whilst maintaining an immunogenic formulation known to give protection. This is particularly important when using adjuvants that contain oil as the presence of both oil and LPS can cause severe septic shock in animals. For this reason, we targeted the isolation and purification of the *B. bronchiseptica* fimbrial antigen. Fimbriae are surface antigens that mediate adhesion of the microorganism to the mucosal epithelial cells. As such, they play a crucial role in initiating and sustaining attachment to the respiratory mucosa. It was therefore hypothesised that immune mediated disruption of their function would reduce the burden of infection and associated clinical disease.

In a series of experiments, we were able to demonstrate that a novel *B. bronchiseptica* vaccine comprising purified fimbrial extracts formulated in a vitamin E-based oil-in-water adjuvant can be given to young puppies and pregnant dams with a high degree of safety. In addition, it was able to provide a high level of protection against clinical signs and bacterial excretion when delivered to 5–6-week-old puppies by a convenient injectable route concurrently with routine vaccines, positioning this vaccine as a uniquely versatile and practical alternative to oral and intranasal *Bordetella* vaccines.

## 2. Materials and Methods

### 2.1. Generation of Fimbrial Antigen Extracts from Bordetella bronchiseptica

*B. bronchiseptica* fimbrial antigen extracts were produced by dissociating the fimbriae from the cell surface, concentrating and purifying them by ultrafiltration, PEG precipitation and SDS extraction. In short, *B. bronchiseptica* strain Bb7 was cultured in Tryptose Phosphate Broth (TPB) (Oxoid, UK) and incubated at 37 °C overnight. The fimbriae were detached by heat shock at 65 °C for a period of 15 min followed by a pH shock at pH 8.2 for a period of 15 min. Subsequently the cells were removed by centrifugation and the supernatant clarified by microfiltration and diafiltration. The fimbriae were concentrated by ultrafiltration and the resulting fimbriae containing fraction concentrated by PEG precipitation and continuous flow centrifugation. The pellet was resuspended and the fimbriae further purified by SDS precipitation and continuous flow centrifugation. The final *B. bronchiseptica* fimbrial antigen harvest was inactivated with chlorocresol.

### 2.2. Vaccine Formulations

A newly developed *B. bronchiseptica* vaccine comprising purified fimbrial extracts formulated in a vitamin E-based oil-in-water adjuvant was used in the studies: Nobivac Respira Bb (MSD Animal Health). For the preparation of vaccine candidates various quantities of purified *B. bronchiseptica* fimbrial antigen were formulated with 74.7 mg (7.5%) dl-α-tocopheryl acetate (Micro Diluvac Forte), 0.15 mg thiomersal, disodium hydrogen phosphate dihydrate, sodium dihydrogen phosphate dihydrate, polysorbate 80 and water for injection. The dose volume was 1 mL, and the vaccines were stored at 2–8 °C until use, when they were warmed to room temperature.

### 2.3. Concurrent Vaccines

Nobivac DHPPi (MSD Animal Health) is a core combination dog vaccine that contains live attenuated canine adenovirus type 2, canine distemper virus, canine parvovirus and canine parainfluenza virus strains.

Nobivac L4 (MSD Animal Health) is an inactivated, non-adjuvanted, pentavalent whole bacterin vaccine containing *L. interrogans* serogroup Canicola serovar Portland-vere, *L. interrogans* serogroup Icterohaemorrhagiae serovar Copenhageni, *L. interrogans* serogroup Australis serovar Bratislava, and *L. kirschneri* serogroup Grippotyphosa serovar Dadas. The vaccines were administered subcutaneously according to manufacturer’s instructions, concurrently with Nobivac Respira Bb to reflect standard veterinary practice.

### 2.4. Challenge Material and Challenge Method

*Bordetella bronchiseptica* strain D-2 (gifted by University of Iowa) was cultured on Tryptose-Phosphate-Broth (TPB) agar plates (Oxoid, UK) incubated at 37 °C overnight. Following incubation, 10 mL fresh TPB broth was added to the surface of each plate. Using a plate spreader, the bacterial growth was suspended in the medium. The culture was centrifuged and the pellet resuspended with fresh TPB broth to obtain counts of at least 5 × 10^9^ CFU/mL. The challenge was administered on two consecutive days and freshly prepared for each day of challenge. On each day, each dog was inoculated with 0.5 mL (2.5 × 10^9^ CFU per inoculation) of a freshly prepared suspension of *B. bronchiseptica* strain D-2 per nostril.

### 2.5. Nasal Swab Isolation and Culture

Nasal swab samples were taken by swabbing both nostrils of the dog and collecting the swab in TPB transport medium. All nasal swab samples were cultured on Bordetella Selective Medium agar plates (Oxoid, UK) following serial dilution in TPB medium and incubated aerobically for 48 h at 37 °C.

### 2.6. Serological Analysis

Serum samples were assayed for antibodies to *Bordetella bronchiseptica* using an enzyme-linked immunosorbent assay (ELISA). The ELISA 96-well plates were coated with purified *Bordetella* fimbrial antigen in carbonate-bicarbonate coating buffer and incubated at 37 °C overnight. The contents of each well were aspirated and blocked with 200 µL per well of blocking buffer (PBS 0.04 M (pH 7.2), 1% BSA, 0.01% thiomersal) and incubated for 30 min at 37 °C. Plates were washed 4 times with distilled water and the pre-diluted test sera added. The test sera were diluted 2-fold across the plate and incubated for one hour at 37 °C. The plates were washed 4 times, and 100 µL Anti-Dog IgG (whole molecule) peroxidase conjugate (Sigma-Aldrich, Germany) was added to all wells at 1 in 10,000 dilution and incubated for 30 min at 37 °C. The plates were washed 4 times, coated in a TMB substrate and incubated for 15 min at room temperature in the dark. The colour reaction was stopped by the addition of 4N sulphuric acid and the absorbances read at a 450 nm optical density. Positive and negative control dog sera were used as test controls. Antibody titres were calculated as the log_2_ dilution of the absorbance (450 nm) value at the intercept of the cutoff (cutoff value calculated per assay as 3 times the average value of the negative control sera OD_450 nm_ value).

Antibodies specific to vaccination with Nobivac DHPPi were measured using haemagglutination inhibition and virus neutralisation assays as previously described [21]. Antibodies specific to vaccination with Nobivac L4 were measured using an in-house microscopic agglutination test (MAT) as previously described [22].

### 2.7. Clinical Monitoring

Post-challenge, all dogs were observed daily for clinical signs until the end of the study. The dogs were monitored for a comprehensive range of clinical parameters, including but not limited to general health (reduced appetite, malaise, dehydration, polydipsia, jaundice, and poor condition), respiratory (hyperpnoea-accentuated breathing, dyspnoea-distressed breathing, coughing, and tracheal palpation), ocular (lachrymation, discharge, and conjunctivitis), nasal (ulcers, discharge, and sneezing), oral (ulcers, excess salivation, and gingivitis), abdominal (ascites), intestinal (vomiting and diarrhoea), and musculoskeletal lameness and central nervous system (twitching, circling, ataxia, convulsions, and paralysis). Clinical signs indicative of *B. bronchiseptica* respiratory infection were scored according to Table 1.

### 2.8. Statistical Analysis

Serological response to vaccination was compared at the titre level by the two-sample t-test and compared to the control group. In addition, a statistical group comparison was performed for the dose response study by One-Way Analysis of Variance (ANOVA) using the Tukey–Kramer adjustment for multiple comparisons. Clinical scores (i.e., spontaneous coughing, coughing on palpation, and total clinical score) post-challenge were analysed as an ordinal response using a cumulative logit model that was fitted by means of Generalised Estimating Equations (GEEs) in order to take into account the repeated measurement structure of the data (SAS proc genmod V9.4, repeated statement). *B. bronchiseptica* excretion post-challenge was represented by the Area Under the Curve (AUC) for the Log10 bacterial counts and were compared between treatment groups using the non-parametrical Kruskal–Wallis test and were pairwise compared to the control group using Wilcoxon’s rank-sum test. A *p*-value of 0.05 (2-sided) was considered significant for all tests.

### 2.9. Animals, Housing and Ethics Statement

All studies were performed in conventional 5 to 6-week-old Beagle puppies of mixed sex from a commercial supplier. During the experiments, puppies were group housed in self-contained rooms, and food and water were available *ad libitum* throughout. All experiments were conducted in accordance with the Animal Health and Welfare regulations and approved by the Animal Welfare Review Board, MSD Animal Health, and were registered according to the UK legislation under project licence PPL 80/2586 in compliance with Directive 2010/63/EU. Prior to the start of each study each animal was examined and declared to be healthy and suitable for inclusion. The animals were continuously monitored under veterinary care.

### 2.10. Experimental Design

#### 2.10.1. Study 1: Dose Response in Naïve 5–6-Week-Old Puppies

**Figure 1:**
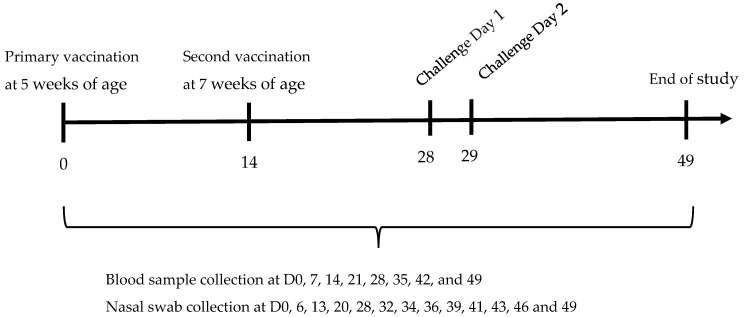
Dose response in naïve 5–6-week-old puppies: Schematic representation of the vaccination and challenge timeline.

Thirty-nine naïve Beagle puppies of 5–6 weeks of age (from 5 litters) were divided into four groups equally spread by litter and sex. Groups 1, 2 and 3 comprised 10 dogs (vaccinated), and group 4 comprised 9 dogs (unvaccinated controls); all groups were mixed across 5 rooms. Groups 1, 2 and 3 were vaccinated subcutaneously with Nobivac Respira Bb formulated to either 69 U, 25 U or 7 U per dose, respectively, followed by a second vaccination two weeks later (day 14). The selection of the highest dose was based on historical experience with medium and lowest doses selected to be sufficiently distinct. This vaccination schedule deviates from the registered schedule in which the second vaccination is given 4 weeks post-primary vaccination; however, during development a 2-week interval was also investigated. All groups were challenged on two consecutive days with *B. bronchiseptica* two weeks after the second vaccination (days 28 and 29). Daily clinical observations were carried out for three weeks post-challenge until the end of the study on day 49. Blood samples were collected from all dogs on days 0, 7, 14, 21 and 28 to determine serological response to vaccination by ELISA and on days 35, 42 and 49 to determine the serological response to challenge. Nasal swabs were collected from all dogs on days 0, 6, 13, 20, and 28 to confirm the pre- and post-vaccination shedding status and on days 32, 34, 36, 39, 41, 43, 46 and 49 to determine relative excretion post-challenge. A schematic representation of the study design is shown in Figure 1.

#### 2.10.2. Study 2: Onset of Immunity in Naïve 5–6-Week-Old Puppies

**Figure 2:**
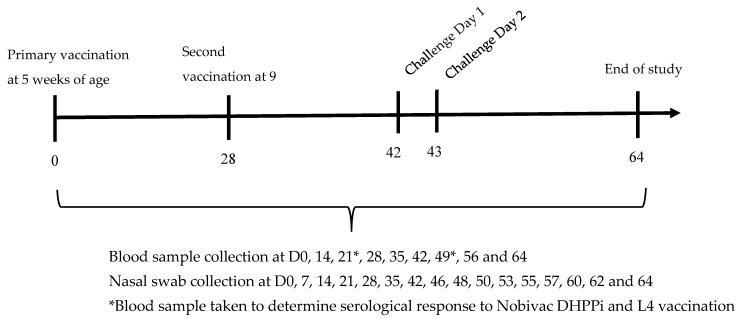
Onset of immunity in naïve 5–6-week-old puppies: Schematic representation of the vaccination and challenge timeline.

Twenty-one naïve Beagle dogs of 5–6 weeks of age were divided into two groups equally spread by litter and sex. Group 1 (vaccinated) comprised 13 dogs, and group 2 (unvaccinated controls) comprised 8 dogs. Dogs in group 1 were vaccinated subcutaneously with Nobivac Respira Bb at a minimum antigen potency of 88 U per dose (selected to ensure duration of immunity extended beyond that of peak immunity), concurrently with Nobivac DHPPi reconstituted with Nobivac L4. A second vaccination with the same vaccines was administered 4 weeks later (day 28). The dogs in group 2 were vaccinated subcutaneously with Nobivac DHPPi reconstituted with Nobivac L4 at the same time as group 1. Two weeks after the second vaccination, the dogs in groups 1 and 2 were challenged with *B. bronchiseptica* (day 42). Daily clinical observations were carried out for three weeks post-challenge until the end of the study on day 64. Blood samples were taken from all dogs on days 0, 7, 14, 21, 28, 35, 42, 49, 56 and 64 to determine serological response to vaccination and challenge. Nasal swabs were collected from all dogs on days 0, 7, 14, 21, 28, 35, and 42 to confirm the pre- and post-vaccination shedding status and on days 46, 48, 50, 53, 55, 57, 60, 62 and 64 to determine relative excretion post-challenge. A schematic representation of the study design is shown in Figure 2.

#### 2.10.3. Study 3: Part 1: Safety in Pregnant Dogs

**Figure 3:**
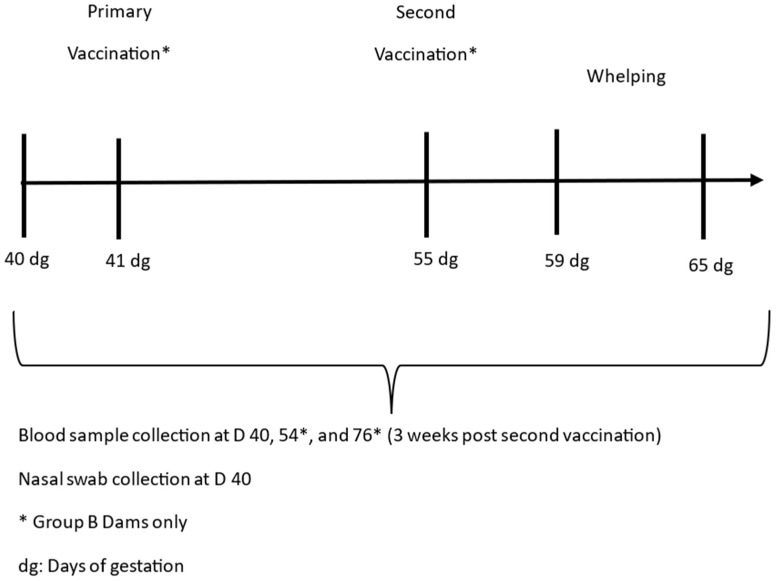
Safety in pregnant dogs: Schematic representation of the vaccination and sampling timeline of the pregnant Beagle dams during gestation (days).

Four pregnant adult Beagle dams were split equally into two groups: A (unvaccinated controls) and B (vaccinated). The group B dogs were vaccinated subcutaneously with Nobivac Respira Bb at a minimum antigen potency of 86 U per dose (selected to ensure duration of immunity extended beyond that of peak immunity) on day 41 of gestation, followed by a second vaccination on day 55 of gestation. Blood was collected from the group A and B dams on day 40 of gestation, and additionally from group B on day 54 of gestation, and at 3 weeks post-second vaccination (day 76) to determine the serological response to vaccination. Nasal swabs were collected from both groups A and B on day 40 of gestation. A schematic representation of the study design is shown in Figure 3.

Part 2: Efficacy in 5–6-week-old puppies positive for maternally derived antibodies

**Figure 4:**
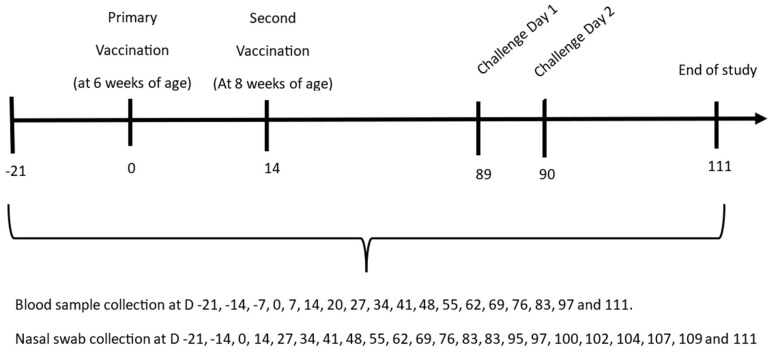
Efficacy in 5–6-week-old puppies positive for maternally derived antibodies: Schematic representation of the vaccination and sampling timeline of the Beagle pups.

Following whelping, the puppies born to the group A and group B dams were allocated to groups based on their maternally derived antibody (MDA) status with respect to *Bordetella*, and their litter group. Group 1 comprised 11 pups (negative for MDAs) whelped from the group A dams; groups 2 and 3 comprised 9 and 8 pups respectively (positive for MDAs) whelped from the group B dams. All pups in group 1 (MDA−) and group 2 (MDA+) were vaccinated at 5–6 weeks of age with a minimum potency formulation of Respira Bb (86 U per dose (selected to ensure duration of immunity extended beyond that of peak immunity)) followed by a second vaccination two weeks later. The pups in group 3 (MDA+) were not vaccinated and controlled for the natural decline in MDAs over time. All puppies were challenged with *B. bronchiseptica* at 19 weeks of age when the MDA levels in group 3 were no longer detectable. Daily clinical observations were made for three weeks post-challenge until the end of the study on day 111. Blood samples were collected from all groups of puppies on days −21, −14, −7, 0, 7, 14, 20, 27, 34, 41, 48, 55, 62, 69, 76, 83, 97 and 111 to determine the serological status. Nasal swabs were collected from the pups on days −21, −14, 0, 14, 27, 34, 41, 48, 55, 62, 69, 76, 83, 83, 95, 97, 100, 102, 104, 107, 109 and 111 to determine the levels of *Bordetella* excretion pre- and post-challenge (Figure 4).

## 3. Results

### 3.1. Study 1: Dose Response in Naïve 5–6-Week-Old Puppies

In order to determine the minimum efficacious dose, vaccines containing *B. bronchiseptica* antigens at 69 (group 1), 25 (group 2), or 7 (group 3) units per dose were formulated in 50% Micro Diluvac Forte containing 7.5% dl-α-tocopheryl acetate in a 1 mL volume. Vaccine efficacy was assessed by challenge with *B. bronchiseptica* two weeks after the second vaccination.

**Figure 5:**
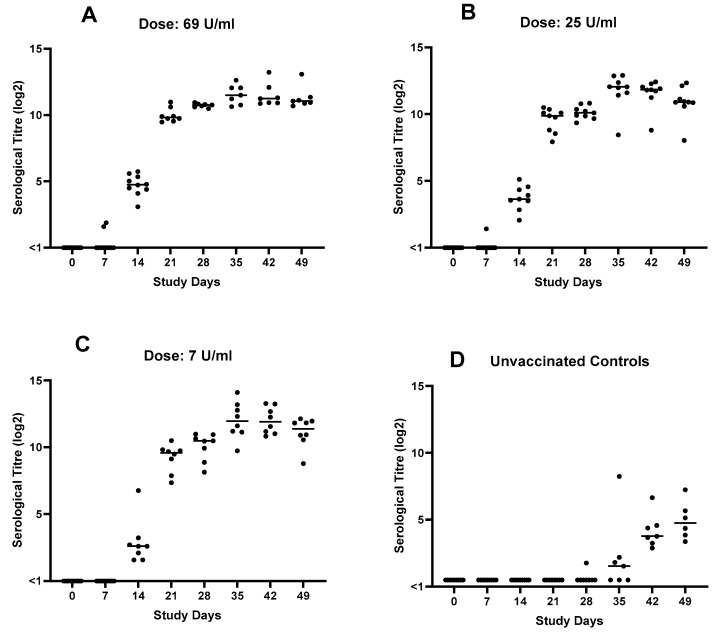
Dose response study: serological titres post-vaccination and post-challenge. Primary vaccination was given on study day 0, and second vaccination was given on study day 14. All dogs were challenged with *B. bronchiseptica* on study day 28. (**A**) Dogs vaccinated with a dose of 69 U/mL, (**B**) dogs vaccinated with a dose of 25 U/mL, (**C**) dogs vaccinated with a dose of 7 U/mL and (**D**) unvaccinated control dogs. Inferential statistics showed statistical significance between groups on day 14 (*p* = 0.0208) with a statistically significant difference between groups 1 and 3 (*p* = 0.0186). Titre < 1 is considered negative.

All pups were seronegative at the start of the study and the unvaccinated group (group 4) remained seronegative throughout the study. Seroconversion was observed in all vaccinated groups between 7 and 14 days post-primary vaccination and titres further increased after the second vaccination. Post-challenge there were marginal rises in serological titres. A dose response relationship post-vaccination was observed, and this was statistically significant between groups on day 14 (*p* = 0.0208), with a statistically significant difference between groups 1 and 3 (*p* = 0.0186). The serological data is shown in Figure 5.

**Figure 6:**
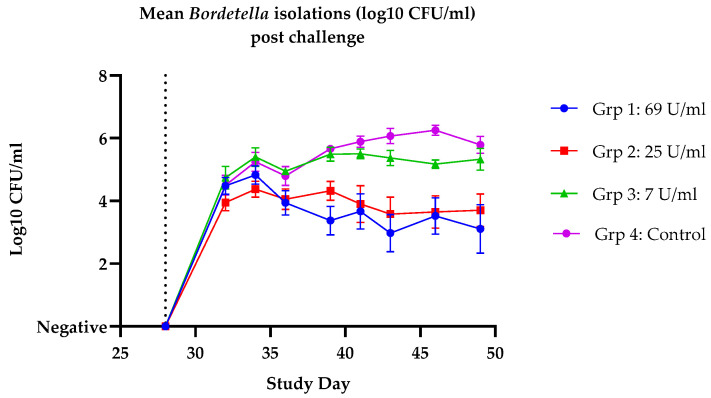
Dose response study: mean *Bordetella bronchiseptica* (Log_10_ CFU/mL) nasal swab isolation post-challenge per group. All dogs were challenged on day 28 (2 weeks post-second vaccination), indicated by the vertical dotted line. Group 1: dogs vaccinated with a dose of 69 U/mL, group 2: dogs vaccinated with a dose of 25 U/mL, group 3: dogs vaccinated with a dose of 7 U/mL and group 4: unvaccinated control dogs. Error bars indicate standard error of the mean (SEM).

Following challenge, clinical signs of a kennel cough infection, characterised by spontaneous coughing and coughing induced by palpation, were of a reduced frequency in the vaccinated dogs when compared with the controls. There was a statistically significant reduction in the total clinical score and in the spontaneous coughing parameter for all three vaccinated groups when compared with the control group. One control dog was euthanised on humane grounds due to a Bordetella infection in the lungs. Post-challenge clinical signs and bacterial isolations from nasal swab samples are shown in Table 2 and Figure 6 respectively. These results demonstrate that all three doses of vaccine tested were able to significantly reduce the clinical signs of *B. bronchiseptica* post-challenge. Furthermore, there was a statistically significant reduction in bacterial re-isolation from both groups 1 (*p* = 0.0350) and 2 (*p* = 0.0360); however, group 3 was not statistically significant (*p* = 0.6282), suggesting that for optimal performance at the onset of immunity 7 U of antigen per dose is insufficient. In the remaining studies a formulation of at least 86 U/dose was used.

### 3.2. Study 2: Onset of Immunity in Naïve 5–6-Week-Old Puppies

To determine the onset of immunity, 5–6-week-old pups were vaccinated with the *B. bronchiseptica* fimbrial antigen at a dose of 88 units formulated in 50% Micro Diluvac Forte containing 7.5% dl-α-tocopheryl acetate in a 1 mL volume. Vaccinations were given concurrently with Nobivac DHPPi resuspended in Nobivac L4, and vaccine efficacy was assessed by challenge with *B. bronchiseptica* two weeks after the second vaccination.

**Figure 7:**
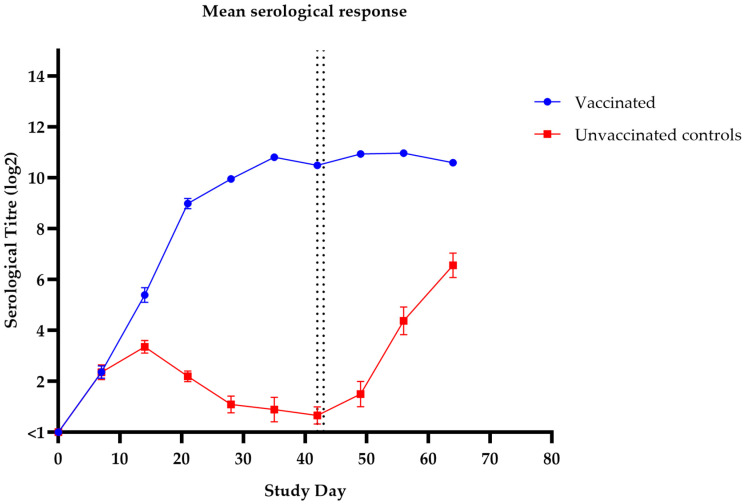
Onset of immunity in naïve 5–6-week-old puppies: mean serological titres post-vaccination and post-challenge. Primary vaccination (V1) was given on study day 0, and second vaccination (V2) was given on study day 28. All dogs were challenged with *B. bronchiseptica* on study days 42 and 43 (indicated by dotted vertical lines). Dogs vaccinated with a dose of 88 U/mL and unvaccinated control dogs. Inferential statistics: significant difference (*p*-value < 0.0001) between groups 1 and 2 on day 42 (just prior to challenge). Error bars indicate standard error of the mean (SEM). Titre < 1 is considered negative.

All pups were seronegative to *Bordetella* before vaccination. Seroconversion was observed in the pups of group 1 at 7 days post-primary vaccination and titres further increased after the second vaccination. The serum antibody titres in the control group were statistically lower than those in the vaccinated group just prior to challenge on day 42 (*p* ≤ 0.0001). Marginal rises in serological titre were observed post-challenge. Low-level antibody titres were detected in the Bordetella ELISA in the unvaccinated control group (group 2) pups prior to challenge. All control pups remained susceptible to challenge and were therefore immunologically naïve to *Bordetella*. The serological data is shown in Figure 7.

**Figure 8:**
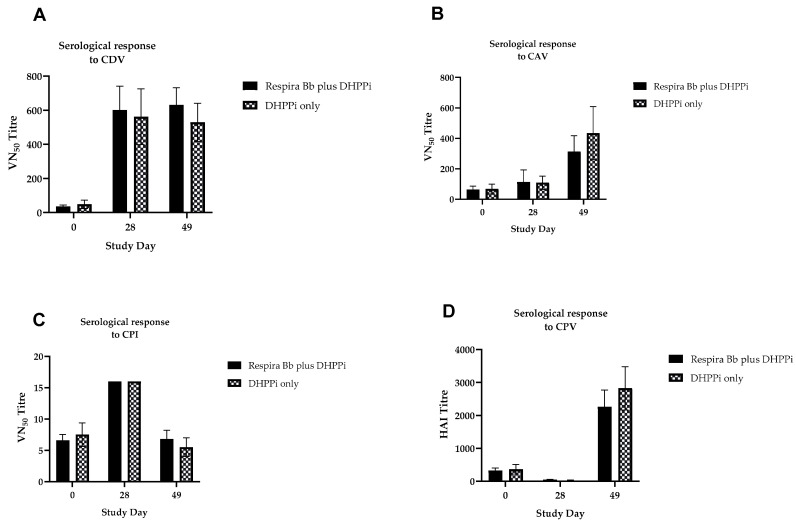
Onset of immunity in naïve 5–6-week-old puppies: mean serological titres post-vaccination with Respira Bb given concurrently with DHPPi, control dogs were vaccinated with DHPPi alone. Primary vaccination (V1) was given on study day 0, and second vaccination (V2) was given on study day 28. All dogs were challenged (**C**) with *B. bronchiseptica* on study day 42, indicated by the dotted vertical line. (**A**) Serological response to CDV, (**B**) mean serological response to CAV, (**C**) mean serological response to Cpi, (**D**) mean serological response to CPV. Error bars represent SEM. No significant difference was observed in antibody titre in dogs vaccinated with DHPPI with and without Respira Bb.

The antibody responses to DHPPi and L4 were not significantly different between the groups throughout the study, demonstrating a lack of interference. The serological data is shown in Figure 8.

Following challenge, the dogs in group 1 developed significantly milder signs of upper respiratory tract disease. In comparison the dogs in group 2 developed clear signs of kennel cough with some severe cases and one dog needing to be euthanised on welfare grounds. These results demonstrate that the *Bordetella* vaccine tested was able to significantly reduce the clinical signs of *Bordetella*-induced disease two weeks post-challenge. Furthermore, there was a statistically significant reduction in *B. bronchiseptica* shedding in the vaccinated group 1 dogs when compared to the unvaccinated group 2 dogs (*p* ≤ 0.0001).

**Figure 9:**
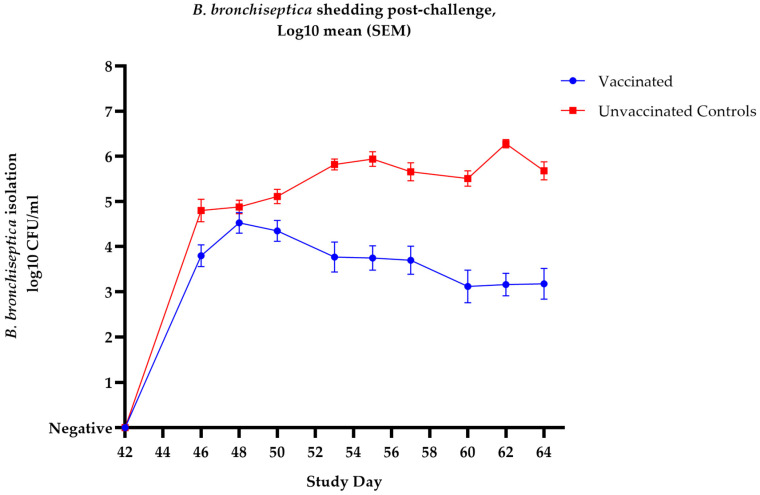
Onset of immunity in naïve 5–6-week-old puppies: mean *B. bronchiseptica* isolations from nasal swabs collected post-challenge following vaccination with Nobivac Respira Bb. Primary vaccination (V1) was given on study day 0, and second vaccination (V2) was given on study day 28. All dogs were challenge with *B. bronchiseptica* on study day 42. Inferential statistics: AUC analysis showed significant difference (*p* ≤ 0.0001) in reduction in *B. bronchiseptica* shedding between vaccinated and unvaccinated control dogs. Error bars represent SEM.

The post-challenge clinical signs and bacterial isolations are shown in Table 3 and Figure 9.

### 3.3. Study 3

Part 1: Safety in pregnant dogs

**Figure 10:**
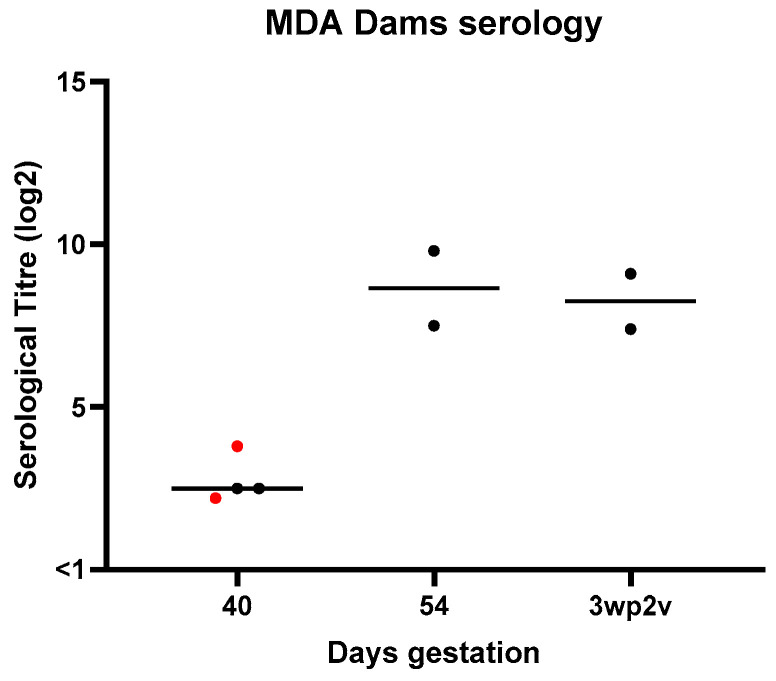
Safety in pregnant dogs: individual serological titres against *B. bronchiseptica* just prior to primary vaccination (40 days), just prior to second vaccination and 3 weeks post-second vaccination (76 days) in pregnant dams vaccinated (black dots) and unvaccinated (red dots) with Nobivac Respira Bb.

To assess safety in pregnancy, four pregnant dams were used, from which two were vaccinated on days 41 and 55 of gestation. All four dams whelped within the expected gestation timeframe and gave birth to healthy puppies, with litter sizes ranging from 6 to 9 pups. One pup was born dead to one of the vaccinated dams from a litter size of 9, which can be expected with the larger litters. Individual dam vaccination schedules and whelping results are shown in Table 4 below. Serological response of pregnant dams pre- and post-whelping is shown in Figure 10.

Part 2: Efficacy in 5–6-week-old puppies positive for maternally derived antibodies

In order to determine the efficacy of the primary vaccination course of Nobivac Respira Bb in 5–6-week-old puppies that were positive for maternally derived antibodies, an MDA− vaccinated group (group 1), an MDA+ vaccinated group (group 2) and an MDA+ unvaccinated control group (group 3) were challenged after monitoring the natural decline in MDAs to determine the optimal point of challenge.

**Figure 11:**
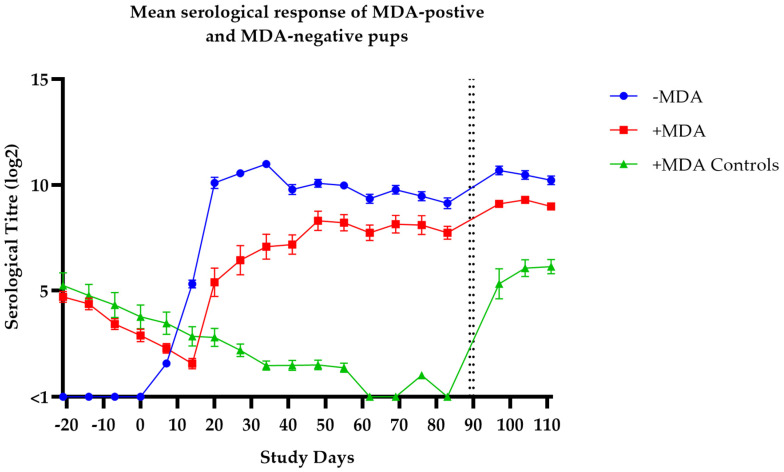
Efficacy in 5–6-week-old puppies positive for maternally derived antibodies: mean serological titres against *B. bronchiseptica* in pups born to vaccinated and unvaccinated dams with Nobivac Respira Bb. Pups vaccinated on day 0 and day 21 followed by *B. bronchiseptica* challenge at days 89 and 90 (indicated by vertical dotted lines). On day 89 (just prior to challenge the antibody titre of both the MDA-positive and MDA-negative vaccinated pups was significantly higher (*p* ≤ 0.0001) than the antibody titre of the MDA-positive non-vaccinated pups. Error bars represent SEM. Titres < 1 are considered negative.

All pups in groups 2 and 3 were seropositive to *Bordetella* prior to vaccination, demonstrating the presence of MDA levels at the point of vaccination. The pups in group 1 remained seronegative to *Bordetella* prior to vaccination. Following vaccination, the pups in group 1 and 2 began to respond between one and three weeks post-second vaccination, and the timing of the group 2 response correlated with the relative MDA levels at vaccination. The pups in group 3 showed a steady decline in MDA levels throughout the study until they were all below the limit of detection on day 83. Prior to challenge the antibody responses in groups 1 and 2 were significantly higher than the controls in group 3 (*p* ≤ 0.0001). Post-challenge there were marginal rises in serological titres in the vaccinated groups and considerable seroconversion in the non-vaccinated group. The serological titres are shown in Figure 11.

**Figure 12:**
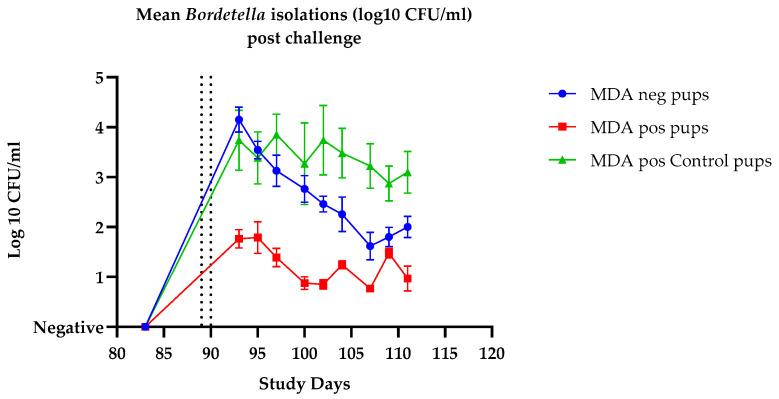
Efficacy in 5–6-week-old puppies positive for maternally derived antibodies: mean *B. bronchiseptica* isolations from nasal swabs collected post-challenge from pups born to Nobivac Respira Bb vaccinated dams (MDA-positive pups) and unvaccinated dams (MDA-negative pups). Pups vaccinated with Nobivac Respira Bb on day 0 and day 21 followed by *B. bronchiseptica* challenge at days 89 and 90 (indicated by vertical dotted lines). Control pups were born to vaccinated dams (MDA-positive) and remained unvaccinated on day 0 and day 21. Error bars represent SEM. Inferential statistics: AUC analysis showed significant difference (*p* = 0.0006) between group 2 vaccinated dogs (MDA+) when compared to the unvaccinated MDA-positive controls (group 3).

After the decline in MDAs in group 3, the puppies in all three groups were challenged intranasally with virulent Bb on two consecutive days with virulent *B. bronchiseptica*. Blood was taken for serology and swabs for isolation as outlined in Figure 4 (line schedule). Post-challenge clinical signs and bacterial isolations are shown in Table 5 and Figure 12 respectively. The pups in groups 1 and 2 showed significantly milder clinical signs compared to controls (*p* ≤ 0.0001) and were not significantly different from each other (*p* = 0.0860). There was a statistically significant reduction in bacterial shedding in the group 2 vaccinated dogs (MDA+) when compared to the group 3 controls (*p* = 0.0006). However, the comparison of bacterial shedding between the group 1 (MDA−) vaccinated dogs and the group 3 controls (MDA+) did not reach statistical significance (*p* = 0.1509). These results demonstrate that primary vaccination of the *B. bronchiseptica* fimbriae at minimum potency in the presence of MDAs significantly reduces the clinical signs and excretion of *Bordetella* post-challenge.

## 4. Discussion

CIRD, caused primarily by *Bordetella bronchiseptica*, is a highly contagious disease occurring not only in dogs housed collectively in shelters or animal hospitals but also following close contact with an affected animal [23]. Alongside *B. bronchiseptica*, viral pathogens such as canine distemper virus, canine parainfluenza virus and canine adenovirus type 2 play a role in this disease complex. Vaccination against these viral components of the CIRD complex forms part of the core vaccination schedule of young puppies providing active immunity against these infectious agents [3]. Whilst a number of different vaccines are available against *B. bronchiseptica*, they differ in their characteristics with regard to the vaccination route, onset of immunity, their ability to reduce clinical signs and shedding of bacteria from infected dogs. Intranasal vaccines are generally considered as very effective vaccines with a rapid onset of immunity from a single dose, which in some cases has been shown to last for over a year [8,24,25]. However, this route of vaccination is not straightforward in some dogs, especially those who are aggressive or anxious. Additionally, sneezing shortly after vaccination can raise concerns about whether the complete dose has been administered and whether or not an additional dose is required. Oral vaccinations may be perceived as more convenient to deliver as less accuracy is needed, but the onset of immunity from these vaccines is usually later than intranasally delivered vaccines though a duration of immunity of one year can also be achieved [8]. In aggressive or anxious dogs these may also be difficult to deliver. Although some owners may prefer these routes of vaccination as they do not involve injections, it is likely that the other core vaccinations given to dogs are injected; thus, in one visit, two different routes of administration, one injection parenterally and one mucosally, would be required.

As alternatives to mucosal delivery there are two *B. bronciseptica* vaccines available which can be injected [6,12]. One is protein extract from *Bordetella bronchiseptica* which is non-adjuvanted, and the other (Pneumodog, Boehringer Ingelheim, Czechia) is an inactivated Bordetella bacterin in combination with canine parainfluenza virus adjuvanted with aluminium hydroxide. However, the full characteristics of these vaccines are unclear especially with regard to age of first use [12], effectivity in the face of maternally derived antibody, concurrent use with core vaccines, duration of immunity and safety in pregnant animals.

The present studies address these gaps by evaluating the safety, onset of immunity and efficacy of a novel injectable *B. bronchiseptica* vaccine in early life, including its performance when administered alongside core canine vaccines and in puppies with maternally derived antibodies. Using a novel and extensive purification process we prepared an extract of *Bordetella bronchiseptica* fimbrial antigen, which at various concentrations, when combined with a proprietary oil-in-water adjuvant based on vitamin E known to be safe in dogs (unpublished data), could induce strong immune responses in vaccinated dogs. We present a comprehensive characterisation of this novel injectable *B. bronchiseptica* fimbrial antigen vaccine across three complimentary studies. The doses tested all showed minimal reactions at the site of injection, with any reactions resolving in a short period as is to be expected with an oil-in-water-based vaccine. Using a dose response approach, we established a minimum dose of antigen, which was able to both give significant clinical protection and significantly reduce bacterial excretion. The onset of immunity was shown to be two weeks after the second vaccination, and whilst this is not as rapid as that following the use of intranasal vaccines, it does give the option to veterinarians to vaccinate via conventional routes concurrently with DHPPi and L4 vaccines without affecting any vaccine efficacy. Low-level antibody titres were detected in the Bordetella ELISA in the control pups, vaccinated with DHPPI +L4, alone prior to challenge. This had been seen previously and is believed to represent a low level of non-specific antibody cross reactivity in the *Bordetella* ELISA following DHPPi + Nobivac L4 administration. All controls remained fully susceptible and therefore immunologically naïve to *Bordetella*.

Another advantage of this vaccine is the age at which vaccination can begin. We have demonstrated that the vaccine is safe and efficacious in puppies at 5–6 weeks of age, whether they have maternally derived antibodies or are antibody-free. This is highly useful considering that vaccination with core vaccines such as distemper, parvovirus, adenovirus-2, parainfluenza virus and leptospira vaccines can be given around this time and via the same route of administration.

## 5. Conclusions

The data presented across these three studies demonstrate that Nobivac Respira Bb (MSD Animal Health) is safe for use in 5–6-week-old puppies alongside other Nobivac core canine vaccines without vaccine interference. The vaccine has an onset of immunity of two weeks and significantly reduces both the clinical signs of *B. bronchiseptica*-induced disease and bacterial excretion into the environment. Furthermore, the vaccine is equally efficacious in puppies with maternally derived antibodies derived from vaccinated dams and can be used safely in pregnant bitches. As such, this vaccine represents a convenient, safe and efficacious alternative to vaccines delivered via the oral or intranasal routes and is a positive addition to the range of vaccines targeted at reducing disease induced by *B. bronchiseptica*.

## Figures and Tables

**Table 1:** Clinical signs indicative of *B. bronchiseptica* respiratory infection and general health observed.

Clinical Signs		Score
Examined in detail, found to be normal		0
Excess lachrymation		1
Cough	2–4 times in 10 min	1
	5–10 times in 10 min	2
Cough on palpation	2–4 times in 10 min	1
	5–10 times in 10 min	2
Diarrhoea, soft mucus		1
Reduced appetite—still eating but delaying going to fresh food (>5 min)		1
Dehydration		4
Malaise/depression and reduced appetite		2
Mild nasal mucopurulent discharge		2
Moribund and euthanasia		150

**Table 2:** Dose response study: summary of clinical observations and scores post-challenge.

		Treatment Group															
Clinical Observation		69 U/mL Dose (*n* = 7)				25 U/mL Dose (*n* = 9)				7 U/mL Dose (*n* = 8)				Control (*n* = 7)			
		Observations			Total Score	Observations			Total Score	Observations			Total Score	Observations			Total Score
		Total	Range	Median		Total	Range	Median		Total	Range	Median		Total	Range	Median	
Cough	2–4 times in 10 min	2	0–2	0	2	8	0–5	0	8	11	0–4	1	11	39	0–14	4	39
	5–10 times in 10 min	0	0	0	0	0	0	0	0	0	0	0	0	2	2	0	4
Cough on palpation	2–4 times in 10 min	20	1–8	2	20	19	0–8	1	19	37	0–13	4	37	73	0–18	9	73
	5–10 times in 10 min	0	0	0	0	0	0	0	0	0	0	0	0	1	1	0	2
Excess lachrymation		2	0–1	0	2	0	0	0	0	20	0–10	0	20	7	0–4	0	7
Moribund and euthanasia		0	0	0	0	0	0	0	0	0	0	0	0	1	0–1	0	150
*p*-value					<0.0001				<0.0001				0.0155				-

The number of observations per clinical sign post-challenge is shown as total, range and median per treatment group. The total clinical score is the number of observations multiplied by the score assigned to the clinical sign. GEE statistical analysis was performed to analyse the sum of the daily clinical scores for all parameters.

**Table 3:** Onset of immunity in naïve 5–6-week-old puppies: Summary of clinical observation and scores post-challenge.

Clinical Observation		Vaccinated Dogs (*n* = 13)				Controls (*n* = 8)			
		Observations			Total Score	Observations			Total Score
		Total	Range	Median		Total	Range	Median	
Cough	2–4 times in 10 min	19	0–5	1	19	73	0–14	10.5	73
	5–10 times in 10 min	0	0	0	0	21	0–6	2	42
Cough on palpation	2–4 times in 10 min	36	0–9	1	36	102	0–18	14.5	102
	5–10 times in 10 min	0	0	0	0	15	0–6	1.5	30
Excess lachrymation		7	0–4	0	7	2	0–1	0	2
Diarrhoea, soft mucus		1	0–1	0	1	0	0	0	0
Reduced appetite		0	0	0	0	1	0–1	0	1
Dehydration		0	0	0	0	1	0–1	0	4
Malaise/depression and reduced appetite		0	0	0	0	1	0–1	0	2
Mild nasal mucopurulent discharge		0	0	0	0	1	0–1	0	2
Moribund and euthanasia		0	0	0	0	1	0–1	0	150
*p*-value					<0.0001				-

The number of observations per clinical sign post-challenge is shown as total, range and median per treatment group. The total clinical score is the number of observations multiplied by the score assigned to the clinical sign. GEE statistical analysis was performed to analyse the sum of the daily clinical scores for all parameters.

**Table 4:** Safety in pregnant dogs: individual dam vaccination and whelping data.

Dam ID	Vaccination Schedule	Whelping Day	Days Post-Second Vaccination That Bitch Whelped	Pups Born	
				Live	Dead
81B	Unvaccinated controls	59	n/a	6	0
B19		65	n/a	5	0
3D11	2 vaccinations two weeks apart on day 41 (23 March 2018) and day 55 (6 April 2018) of gestation	62	7	8	1
12C		64	9	9	0

**Table 5:** Efficacy in 5–6-week-old puppies positive for maternally derived antibodies: summary of clinical observations and scores post-challenge.

Clinical Observation		Treatment											
		MDA-Negative Vaccinated Pups (*n *= 11)				MDA-Positive Vaccinated Pups (*n* = 7)				MDA-Positive Control Non-Vaccinated Pups (*n *= 7)			
		Observations			Total Score		Observations		Total Score		Observations		Total Score
		Total	Median	Range		Total	Median	Range		Total	Median	Range	
Cough	2–4 times in 10 min	3	0	0–1	3	1	0	0–1	1	28	4	0–6	28
	5–10 times in 10 min	0	0	0	0	0	0	0	0	15	1	0–5	30
Cough on palpation	2–4 times in 10 min	38	3	0–12	38	3	0	0–3	3	130	19	0–22	130
	5–10 times in 10 min	0	0	0	0	1	0	0–1	2	3	0	0–2	6
Excess lachrymation		0	0	0	0	0	0	0	0	0	0	0	0
Moribund and euthanasia		0	0	0	0	0	0	0	0	0	0	0	0
*p*-value					<0.0001				<0.0001				-

The number of observations per clinical sign post-challenge is shown as total, range and median per treatment group. The total clinical score is the number of observations multiplied by the score assigned to the clinical sign. GEE statistical analysis was performed to analyse the sum of the daily clinical scores for all parameters.

## Data Availability

All data supporting the findings of this study are included within the manuscript.
